# CD137 expression in cancer cells: regulation and significance

**DOI:** 10.1186/s40880-019-0419-z

**Published:** 2019-11-08

**Authors:** Christophe Glorieux, Peng Huang

**Affiliations:** State Key Laboratory of Oncology in South China, Collaborative Innovation Center for Cancer Medicine, Sun Yat-sen University Cancer Center, Guangzhou, 510060 Guangdong P. R. China

Evasion of immune surveillance is an important feature of cancer, which expresses certain molecules that affect immune functions such as immune checkpoints [1]. Among the molecules that impact the interactions between cancer and immune cells, PD-L1 (programmed death-ligand 1) and CTLA-4 (cytotoxic T-lymphocyte-associated protein 4) are well-known to modulate the immune system and play important roles in cancer development. The tumor necrosis factor receptor superfamily member 9 (TNFRSF9), also known as CD137 or 4-1BB, is another important immune-modulating molecule known to be expressed on the surface of certain immune cells. CD137 was originally discovered in 1989 and reported as a cell surface protein mainly located on activated T cells [2]. Interaction of CD137 with its ligand (CD137L, also known as TNFSF9 or 4-1BBL) on activated antigen-presenting cells (APCs) could lead to bidirectional activation that promotes immunity against cancer [3]. For instance, it has been shown that signals through the CD137 receptor could lead to T cell activation and survival [3]. Reversely, engaged CD137L could impact its expressing cells, such as dendritic cells, leading to their activation and maturation [4]. Hence, the use of agonistic CD137 antibodies such as urelumab and utomilumab is considered as a promising immunotherapeutic approach to treat various types of tumors [5].

Although the expression of CD137 and its functional role in immune cells have been intensively studied, only limited information is available regarding the expression of CD137 in tumor cells. Several reports have described the expression of CD137 in various types of malignancies including lung cancer [6], leukemia [7], and lymphoma [8]. However, the regulatory mechanisms and the significance of CD137 expression in cancer cells remain largely unknown. A recent study has shown that CD137 expression in pancreatic cancer cells might be positively regulated by oncogenic K-ras [9]. Using a K-ras-inducible cell system and cancer cell lines with various K-ras status, the authors demonstrated that K-ras could induce CD137 expression through mitogen-activated protein kinases (MAPK) and NF-κB signaling pathways. This is consistent with the roles of these two signaling pathways in regulating CD137 expression in immune cells [10, 11]. Because activating mutations in K-ras is frequently observed in pancreatic cancer, lung, and colorectal cancer, it seems likely that CD137 expression might be a frequent event in these cancer types.

For cancer cells with activating K-ras mutations, the MAPK signaling and NF-κB pathway seem to function in parallel to promote CD137 expression. Although both pathways are driven by K-ras activation, each pathway appears to operate independently and is likely able to compensate each other if one of them is suppressed. The NF-κB pathway seems mainly activated by extracellular IL-1α (interleukin-1 alpha), whose expression and secretion are enhanced by K-ras [12]. In contrast, the MAPK pathway is stimulated by K-ras activation without the involvement of IL-1α [9]. It is currently unclear how the activation of K-ras could lead to increased expression of IL-1α, or which transcription factor(s) might bind and regulate the *CD137* transcription in cancer cells. The relative contributions of the MAPK and NF-κB pathways in promoting the CD137 expression in cancer are likely cell type-dependent, as evinced by the findings that specific suppression of each pathway affected CD137 expression differently in different cell types [9]. Both pathways could be active in certain cancer cells, while in other cancer cells one of the pathways could be dominant. An interesting finding was that IL-1α could stimulate the expression of CD137 in multiple cancer cell lines, whereas neutralization of the IL-1α by antibody failed to decrease the CD137 expression in most of these cell lines [9]. This suggests that these cancer cells might have more than one pathway to promote CD137 expression and thus are not exclusively dependent on IL-1α for stimulation. As such, the design of immunomodulation using IL-1α antibodies should consider this complexity.

The expression of CD137 in immune cells and its role in promoting immune function against cancer have been well-characterized. In fact, CD137 agonists have been tested as anticancer agents with some success [5]. However, the biological significance of CD137 expression in cancer still remains unclear. In leukemia and lymphoma, CD137 expression could promote the growth and survival of malignant cells and inhibit T cell activation [7, 8], suggesting that the overall effect of CD137 expression in cancer cells might be immunosuppressive. It is unclear, however, if such an immunosuppressive function could also be seen in solid tumors. Furthermore, the impact of CD137 expression in cancer cells on the tumor microenvironment may be difficult to predict. On one hand, the expression of CD137 on the cancer cell surface could potentially function as a competitor of CD137 on T cells to bind CD137L on the surface of APCs, and thus suppressing the immune responses against cancer. On the other hand, the interaction of CD137 on the cancer cell surface with CD137L on APCs could potentially activate or promote immune responses. Obviously, further studies are needed to differentiate whether tumor CD137 expression will stimulate or inhibit the functions of immune cells.

Since CD137 is an important molecule known to be expressed in immune cells and modulates immune functions, the discovery that K-ras-driven cancer cells also express CD137 should prompt further investigation in this area. The important questions that remain to be answered are: (1) what is the overall impact of CD137 expression in tumor cells on immune functions against cancer? This question could be addressed using genetic approaches and tumor formation study in immuno-competent animal models; (2) what are the specific effect of tumor CD137 on the functions of APC, T cells, or macrophage? (3) In addition to K-ras, could other oncogenes such as c-Myc and Bcr-Abl regulate the expression of CD137 expression in cancer cells? If so, what are the regulatory mechanisms? The answers to these questions would provide important new information on the role of CD137 in cancer immunity with potential clinical implications.

## Data Availability

Not applicable.

## Abbreviations

ABL: abelson murine leukemia viral oncogene homolog 1
AP-1: activator protein 1
APCs: antigen-presenting cells
BCR: breakpoint cluster region protein
CD137/TNFRSF9: tumor necrosis factor receptor superfamily member 9
CD137L/TNFSF9: tumor necrosis factor ligand superfamily member 9
CTLA-4: cytotoxic T-lymphocyte-associated protein 4
IL: interleukin
K-ras: V-Ki-ras2 Kirsten rat sarcoma viral oncogene homolog
MAPK: mitogen-activated protein kinase
NF-κB: nuclear factor kappa-light-chain-enhancer of activated B cells
PD-L1: programmed death-ligand 1

## References

[1] Larsen TV, Hussmann D, Nielsen AL (2019). PD-L1 and PD-L2 expression correlated genes in non-small-cell lung cancer. Cancer Commun (Lond)..

[2] Kwon BS, Weissman SM (1989). cDNA sequences of two inducible T-cell genes. Proc Natl Acad Sci USA..

[3] Wang C, Lin GH, McPherson AJ, Watts TH (2009). Immune regulation by 4-1BB and 4-1BBL: complexities and challenges. Immunol Rev..

[4] Shao Z, Schwarz H (2011). CD137 ligand, a member of the tumor necrosis factor family, regulates immune responses via reverse signal transduction. J Leukoc Biol..

[5] Chester C, Sanmamed MF, Wang J, Melero I (2018). Immunotherapy targeting 4-1BB: mechanistic rationale, clinical results, and future strategies. Blood..

[6] Zhang GB, Dong QM, Hou JQ, Ge Y, Ju SG, Lu BF (2007). Characterization and application of three novel monoclonal antibodies against human 4-1BB: distinct epitopes of human 4-1BB on lung tumor cells and immune cells. Tissue Antigens..

[7] Palma C, Binaschi M, Bigioni M, Maggi CA, Goso C (2004). CD137 and CD137 ligand constitutively coexpressed on human T and B leukemia cells signal proliferation and survival. Int J Cancer..

[8] Ho WT, Pang WL, Chong SM, Castella A, Al-Salam S, Tan TE (2013). Expression of CD137 on Hodgkin and Reed-Sternberg cells inhibits T-cell activation by eliminating CD137 ligand expression. Can Res..

[9] Glorieux C, Huang P (2019). Regulation of CD137 expression through K-Ras signaling in pancreatic cancer cells. Cancer Commun (Lond)..

[10] Kim JD, Kim CH, Kwon BS (2011). Regulation of mouse 4-1BB expression: multiple promoter usages and a splice variant. Mol Cells..

[11] Kim JO, Kim HW, Baek KM, Kang CY (2003). NF-kappaB and AP-1 regulate activation-dependent CD137 (4-1BB) expression in T cells. FEBS Lett..

[12] Wang L, Bi XW, Zhu YJ, He YZ, Lai QY, Xia ZJ (2018). IL-2Ralpha up-regulation is mediated by latent membrane protein 1 and promotes lymphomagenesis and chemotherapy resistance in natural killer/T-cell lymphoma. Cancer Commun (Lond)..

